# Survivorship care for people affected by advanced or metastatic cancer: MASCC-ASCO standards and practice recommendations

**DOI:** 10.1007/s00520-024-08465-8

**Published:** 2024-04-29

**Authors:** Nicolas H. Hart, Larissa Nekhlyudov, Thomas J. Smith, Jasmine Yee, Margaret I. Fitch, Gregory B. Crawford, Bogda Koczwara, Fredrick D. Ashbury, Maryam B. Lustberg, Michelle Mollica, Andrea L. Smith, Michael Jefford, Fumiko Chino, Robin Zon, Meera R. Agar, Raymond J. Chan

**Affiliations:** 1 Human Performance Research Centre, INSIGHT Research Institute, Faculty of Health, University of Technology Sydney (UTS), Sydney, NSW Australia; 2 Caring Futures Institute, College of Nursing and Health Science, Flinders University, Adelaide, SA Australia; 3 Exercise Medicine Research Institute, School of Medical and Health Science, Edith Cowan University, Perth, WA Australia; 4 Cancer and Palliative Care Outcomes Centre, School of Nursing, Faculty of Health, Queensland University of Technology (QUT), Brisbane, QLD Australia; 5 Institute for Health Research, The University of Notre Dame Australia, Perth, WA Australia; 6 Internal Medicine, Brigham and Women’s Hospital, Harvard Medical School, Boston, MA USA; 7 Division of General Internal Medicine and Sidney Kimmel Comprehensive Cancer Center, John Hopkins Medical Institutions, Baltimore, MD USA; 8 Centre for Medical Psychology and Evidence-Based Decision-Making, Faculty of Medicine and Health, University of Sydney, Sydney, NSW Australia; 9 School of Graduate Studies, Faculty of Nursing, University of Toronto, Toronto, ON Canada; 10 Faculty of Health and Medical Sciences, University of Adelaide, Adelaide, SA Australia; 11 Northern Adelaide Local Health Network, Adelaide, SA Australia; 12 Flinders Health and Medical Research Institute, College of Medicine and Public Health, Flinders University, Adelaide, SA Australia; 13 Flinders Cancer and Innovation Centre, Flinders Medical Centre, Adelaide, SA Australia; 14 VieCure, Clinical and Scientific Division, Greenwood Village, CO USA; 15 Department of Oncology, University of Calgary, Calgary, ON Canada; 16 Internal Medicine-Medical Oncology, College of Medicine, The Ohio State University, Columbus , OH USA; 17 Department of Medicine, School of Medicine, Yale University, New Haven, CT USA; 18 Medical Oncology Division, Yale Cancer Centre, New Haven, CT USA; 19 Office of Cancer Survivorship, Division of Cancer Control and Population Sciences, National Cancer Institute, Bethesda, MD USA; 20 The Daffodil Centre and University of Sydney: a joint venture with Cancer Council NSW, Sydney, NSW Australia; 21 Australian Cancer Survivorship Centre, Peter MacCallum Cancer Centre, Melbourne, VIC Australia; 22 Sir Peter MacCallum Department of Oncology, University of Melbourne, Melbourne, VIC Australia; 23 Department of Radiation Oncology, Memorial Sloan Kettering Cancer Center, New York, NY USA; 24 Michiana Hematology-Oncology, Mishawaka, IN USA; 25 Cincinnati Cancer Advisors, Norwood, OH USA; 26 IMPACCT Research Centre, Faculty of Health, University of Technology Sydney (UTS), Sydney, NSW Australia

**Keywords:** Survivorship, Supportive Care, Elements of Care, Cancer Survivors, Caregivers

## Abstract

**Purpose:**

People with advanced or metastatic cancer and their caregivers may have different care goals and face unique challenges compared to those with early-stage disease or those nearing the end-of-life. These MASCC-ASCO standards and practice recommendations seek to establish consistent provision of quality survivorship care for people affected by advanced or metastatic cancer.

**Methods:**

An expert panel comprising MASCC and ASCO members was formed. Standards and recommendations relevant to the provision of quality survivorship care for people affected by advanced or metastatic cancer were developed through conducting: (1) a systematic review of unmet supportive care needs; (2) a scoping review of cancer survivorship, supportive care, and palliative care frameworks and guidelines; and (3) an international modified Delphi consensus process.

**Results:**

A systematic review involving 81 studies and a scoping review of 17 guidelines and frameworks informed the initial standards and recommendations. Subsequently, 77 experts (including 8 people with lived experience) across 33 countries (33% were low-to-middle resource countries) participated in the Delphi study and achieved ≥ 94.8% agreement for seven standards (1. Person-Centred Care; 2. Coordinated and Integrated Care; 3. Evidence-Based and Comprehensive Care; 4. Evaluated and Communicated Care; 5. Accessible and Equitable Care; 6. Sustainable and Resourced Care; 7. Research and Data-Driven Care) and ≥ 84.2% agreement across 45 practice recommendations.

**Conclusion:**

Standards of survivorship care for people affected by advanced or metastatic cancer are provided. These MASCC-ASCO standards will support optimization of health outcomes and care experiences by providing guidance to stakeholders in cancer care (healthcare professionals, leaders, and administrators; governments and health ministries; policymakers; advocacy agencies; cancer survivors and caregivers. Practice recommendations may be used to facilitate future research, practice, policy, and advocacy efforts.

**Supplementary Information:**

The online version contains supplementary material available at 10.1007/s00520-024-08465-8.

## Introduction

There is an emerging population of people with advanced or metastatic cancer [1, 2] (i.e., solid or hematological malignancies that are treatable yet predominantly incurable [3, 4]) who are likely to have different care goals and face unique care challenges compared to those with early-stage or localized disease or those nearing the end-of-life [5–7]. The complex nature of advanced or metastatic cancers and their treatment sequelae is exemplified by varied disease trajectories that includes periods of disease stability, disease progression with the potential for further therapy, and possibility of sudden transition to end-of-life care [7, 8]. Significantly higher physical, financial, spiritual, and psychosocial symptom burden results, with advanced or metastatic cancer survivors and caregivers thus engaging with the healthcare system at a higher frequency and intensity than those with early-stage disease [8, 9]. Despite continually improving outcomes, people with advanced or metastatic cancer may be intentionally or inadvertently denied quality survivorship care that has become emphasized for those living with and beyond curable cancers. These individuals may therefore not be offered (or may feel excluded from) survivorship services within settings that have finite resources, and/or those that may not feel equipped to provide such services [10]. Additionally, these individuals also face high uncertainty about their future, as well as stigma and discrimination associated with the advanced nature of their disease [7], requiring unique services and targeted resources for their needs. Given the diverse unmet needs of advanced or metastatic cancer survivors and their caregivers [4] across psychological, physical, daily living, financial, health system information, as well as care and support domains, there is an increasing demand for high-quality survivorship care to meet the unique needs of this growing population [5, 6]. While several guidance documents exist to improve cancer survivorship care, these emphasise the post-treatment phase [11–15] and do not address the unique needs of people affected by advanced or metastatic cancer. Furthermore, while priority indicators of quality care for adolescents and young adults with advanced cancer have recently been developed [16], these are not transcendent to the broader advanced or metastatic cancer population, do not include caregivers, and are not focused on survivorship care.

There have been recent calls to advance clinical care, research, policy, and practice in advanced or metastatic cancer survivorship, as well as the establishment of key priority areas to improve care and outcomes for cancer survivors and caregivers [5–8]. In 2011, the American Society of Clinical Oncology (ASCO) released a statement [17] advocating for an individualized approach to clinical discussions and the provision of care for people affected by advanced cancer, emphasizing the need for improved communication, disease-directed and supportive care options, and further research in advanced cancer care. Years later, in 2019, Langbaum and Smith [5] highlighted the urgent need for researchers to address the social, psychological, spiritual, and financial impact of survivorship for those with incurable cancer and called for the focused study of metastatic cancer survivorship. The National Cancer Institute (NCI) identified gaps and opportunities for targeted survivorship research among advanced and metastatic cancers, including the development and assessment of purpose-built models of comprehensive survivorship care [6]. In alignment with this, Smith and colleagues [7] called for research within advanced or metastatic cancer survivorship to ensure survivorship services, models of care, and guidelines are diverse and inclusive, rather than only for those who have completed curative-intent treatment. Smith and colleagues [7] further emphasised the importance of recognizing the complexity of the supportive care needs of those with advanced or metastatic cancer and their caregivers, including the extensive need for psychosocial and practical care. This year, Lai-Kwon and colleagues [8] mapped metastatic cancer survivorship to clinical care, policy, and research domains, providing six themes canvassing advanced or metastatic cancer, from recommendations by the 2006 Institute of Medicine (IOM) landmark report, *From Cancer Patient to Cancer Survivor: Lost in Transition* [18] and 2013 report, *Delivering high-quality cancer care: charting a new course for a system in crisis* [19]: (1) advocacy and policy, (2) communication and care coordination, (3) system capacity and healthcare delivery (4) defining, measuring, and managing quality, (5) addressing inequality, and (6) research. Lai-Kwon and colleagues [8] concluded that current survivorship care systems are not designed to provide optimal care for people affected by advanced or metastatic cancers. Thus, developing standards of quality survivorship care for advanced or metastatic cancer survivors and their caregivers with broad stakeholder engagement and actionable practice recommendations is a priority [6–8].

The aim of this joint effort between the Multinational Association for Supportive Care in Cancer (MASCC) and ASCO was to develop standards for quality survivorship care of people affected by advanced or metastatic cancer. The standards seek to support the optimization of care experiences and health outcomes for these people by providing core recommendations to key stakeholders, including healthcare professionals, hospital and health service administrators, governments, policymakers, and cancer survivors and caregivers that are expected to appropriately inform clinical care, research, policy, and advocacy around cancer survivorship care for those with advanced or metastatic cancer.

## Methods

### Standards development process

These standards and practice recommendations were developed by a multidisciplinary and international Expert Panel from MASCC and ASCO broadly representing various disciplines of cancer care, including cancer specialists (oncologists, hematologists, oncology nurses), palliative care physicians, primary care physicians, allied health professionals, organizational leaders, and distinctly sought and incorporated the perspectives of people with lived experience of advanced or metastatic cancer. Specifically, these standards of quality survivorship care for people affected by advanced or metastatic cancer and practice recommendations were developed through (1) a systematic review of the unmet supportive care needs for people with advanced or metastatic cancer and their caregivers; (2) a scoping review of prominent cancer survivorship, palliative care, and supportive care frameworks and guidelines by leading cancer organisations worldwide; and (3) the completion of an international modified Delphi consensus process.

### Systematic and scoping review

Standards and practice recommendations were initially developed through a synthesis of literature including a high-quality systematic review [4] of unmet needs for people affected by advanced or metastatic cancer that was conducted by experts from MASCC and ASCO. This systematic review included 85 papers representing 81 unique studies across solid tumors (37%; n = 30), hematological malignancies (25%; n = 20), mixed or unspecified cancer types (38%, n = 31) involving cancer survivors and caregivers. In addition, a scoping review was conducted across 17 key cancer survivorship and palliative care frameworks, guidelines, or statements published by leading cancer organisations, including ASCO, Canadian Cancer Research Alliance, Cancer Australia, Clinical Oncology Society Australia, European Society of Medical Oncology, Lancet Oncology Commission, LIVEStrong Essential Elements of Survivorship Care, National Academy of Medicine, National Comprehensive Cancer Network, National Cancer Center Japan, National Cancer Institute, and Palliative Care Australia [6, 11–15, 17, 20–30].

### Establishing consensus

**Fig. 1:**
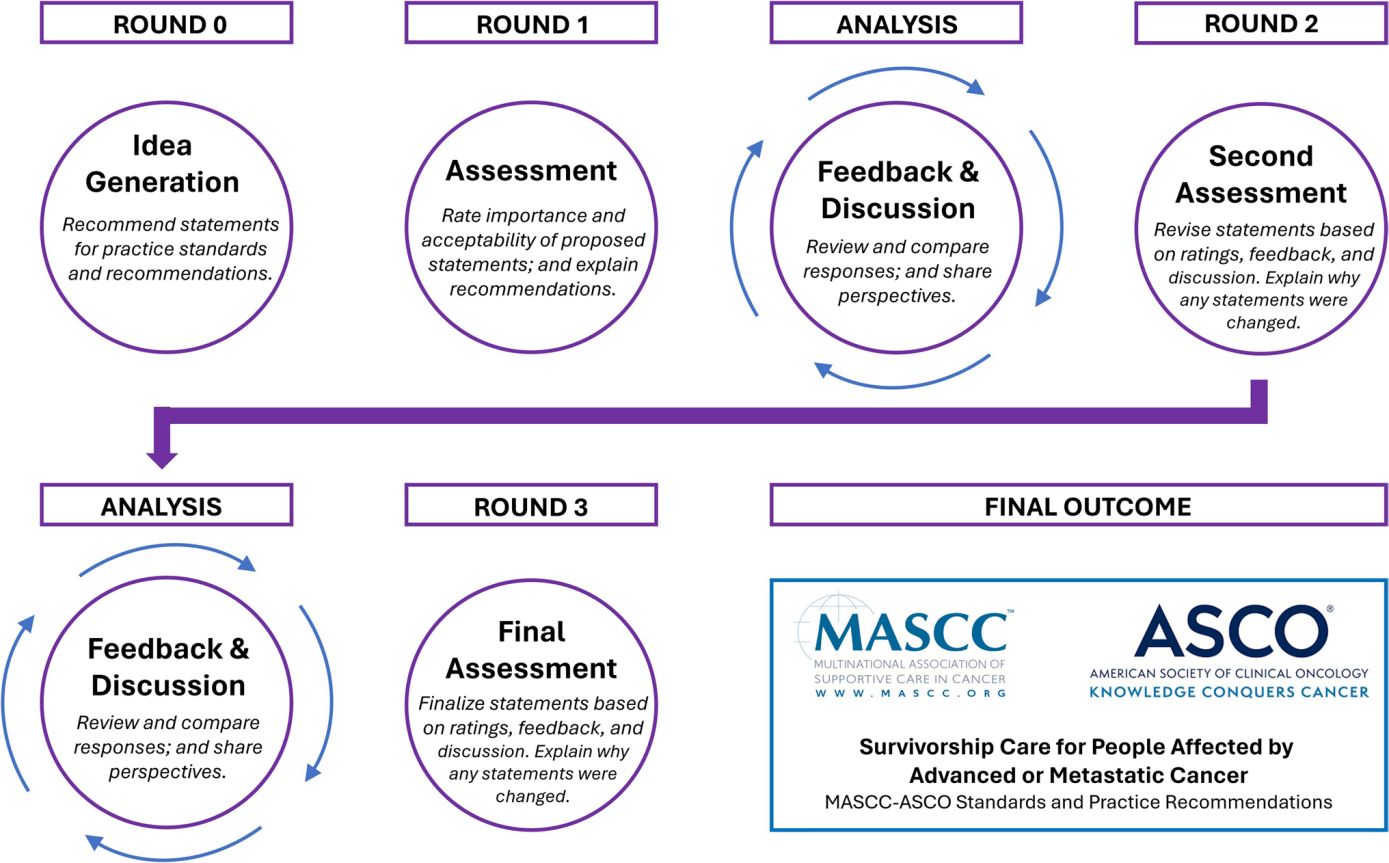
Modified online Delphi process conducted through multiple rounds of idea generation, assessment, feedback, discussion, and presentation of the final MASCC-ASCO Standards and Practice Recommendations for Advanced or Metastatic Cancer

International experts in the field of cancer care for people with advanced or metastatic cancer were identified through MASCC and ASCO membership, and through snowball recruitment by MASCC and ASCO Expert Panel, ensuring representation across clinical areas of expertise and the inclusion of patient advocates, with an a priori recruitment target of 60 participants globally with consideration of over- or under-representation of disciplines, countries, and cancer survivors or caregivers. A modified online Delphi method was used to capture levels of agreement through a structured consensus process (Figure 1) ﻿that systematically used high-quality literature, and opinion of key stakeholders to reach levels of agreement (a priori ≥ 80% of agree or strongly agree). Ethics approval was provided by the Flinders University Human Research Ethics Committee (ID: 4999-HART). Informed consent was obtained from all study participants.

### Harmonizing definitions

These MASCC-ASCO standards have been developed for multinational implementation by a global audience and stakeholders worldwide. It was therefore important to ensure unified definitions and terminology were adopted by the MASCC-ASCO Expert Panel, as well as the international experts recruited to the Delphi consensus process.

Advanced cancer was broadly defined as any solid or hematological malignancy unlikely to be cured or controlled with treatment [3, 4], whereas metastatic cancer was defined as the de novo or recurrent diagnosis of any cancer where the primary tumor has spread beyond its origin to a distant organ or tissue [8, 31], recognizing these are not mutually exclusive terms (i.e., some cancers may be advanced but not metastatic as is the case among many hematological and central nervous system malignancies; some cancers may be metastatic but not advanced as is often the case for metastatic testicular cancer and potentially metastatic melanoma treated with curative intent; and some cancers may be both advanced and metastatic such as metastatic prostate or breast cancer). These definitions were required as no commonly agreed paradigm exists for some advanced cancers owing to their vast heterogeneity of diseases, treatment options, outcomes, disease trajectories, and life expectancies [4, 32].

For these MASCC-ASCO standards of survivorship care, it was acknowledged that survivorship parallels between advanced or metastatic cancers exist, regardless of whether diagnosed de novo or as a result of recurrence or disease progression (i.e., advanced or metastatic cancers can have indolent disease trajectories; periods of disease stability; periods of disease progression with the potential for further treatment; and sudden transitions to end-of-life care [4, 8]), and are therefore also congruent with the advanced or metastatic phase of cancer survivorship [6] as defined by the NCI [see Supplementary File 1]. Supportive care was defined in accordance with MASCC, as care provision that seeks to prevent and manage adverse effects of cancer and treatment, including the management of physical and psychological symptoms and side effects across the cancer continuum, from diagnosis through treatment to post-treatment care, with the aim to improve the quality of rehabilitation, secondary cancer prevention, survivorship, and palliative/end-of-life care [33]. As such, palliative/end-of-life care was also defined in accordance with MASCC as a specialist subset of supportive care that seeks to address serious health-related suffering due to the presence of severe illness, especially for those near the end of life [33, 34].

### Standards and conflicts of interest

MASCC-ASCO’s Expert Panel was assembled in accordance with ASCO’s Conflict of Interest Policy Implementation for Clinical Practice Guidelines (“Policy,” found at https://www.asco.org/guideline-methodology). All members of the Expert Panel (as authors of the standards) completed ASCO’s disclosure form, which requires disclosure of financial and other interests, including relationships with commercial entities that are reasonably likely to experience direct regulatory or commercial impact because of promulgation of the developed standards. Categories for disclosure include employment; leadership; stock or other ownership; honoraria, consulting or advisory role; speakers’ bureau; research funding; patents, royalties, other intellectual property; expert testimony; travel, accommodations, expenses; and other relationships. In accordance with the Policy, most Expert Panel members did not disclose any relationships constituting a conflict under the policy.

### Standards disclaimer

The Standards published herein are provided by the Multinational Association for Supportive Care in Cancer (MASCC) and the American Society of Clinical Oncology Inc. (ASCO) to assist providers in clinical decision making. The information herein should not be relied upon as being complete or accurate, nor should it be considered as inclusive of all proper treatments or methods of care or as a statement of the standard of care. With the rapid development of scientific knowledge, new evidence may emerge between the time information is developed and when it is published or read. The information is not continually updated and may not reflect the most recent evidence. The information addresses only the topics specifically identified therein and is not applicable to other interventions, diseases, or stages of disease. This information does not mandate any particular course of medical care. Further, the information is not intended to substitute for the independent professional judgment of the treating provider, as the information does not account for individual variation among patients. MASCC and ASCO provide this information on an “as is” basis and make no warranty, express or implied, regarding the information. MASCC and ASCO specifically disclaim any warranties of merchantability or fitness for a particular use or purpose. MASCC and ASCO assumes no responsibility for any injury or damage to persons or property arising out of or related to any use of this information, or for any errors or omissions.

## Results

A total of 77 participants (> 10% were people with lived experience) across 33 countries (33% were low-to-middle resource countries) achieved ≥ 94.8% agreement for seven standards and ≥ 84.2% agreement across 45 practice recommendations. Demographic characteristics of participants in are presented in Supplementary File 2, with levels of agreement for each standard and practice recommendation are presented in  Supplementary File 3. The full list of MASCC-ASCO Standards and Practice Recommendations are provided in Table 1 (The Bottom Line).

**Table 1:** The Bottom Line

THE BOTTOM LINE
**Survivorship Care for People Affected by Advanced or Metastatic Cancer: MASCC-ASCO Standards and Practice Recommendations**
**Research Question**
What are the standards for quality advanced or metastatic cancer survivorship care?
**Target Population**
People affected by advanced or metastatic cancer (i.e., cancer survivors and caregivers)
**Target Audience**
(a) Healthcare professionals across disciplines, (b) healthcare leaders, administrators, and management, (c) advanced and metastatic cancer survivors and caregivers, and (d) governments, health ministries, and policymakers
**Methods**
An Expert Panel was convened to develop standards based on: (1) a systematic review of unmet needs, (2) a scoping review of cancer survivorship, supportive care, and palliative care frameworks/guidelines, and (3) an international modified Delphi consensus process
**Quality Survivorship Care Standards and Practice Recommendations**
***1. Person-Centered Care***
To recognize people affected by advanced or metastatic cancer as individuals with agency and partners in cancer care, who are served by, and participate in, trusted health systems that respond to their unique needs (e.g., physical, psychosocial, health systems, information, financial, fertility, sexual, spiritual, and relationships) in humane and holistic ways in collaboration with health practitioners and health care organizations in the public, private, and not-for-profit health and related sectors
** People affected by advanced or metastatic cancer (i.e., cancer survivors, caregivers, and family members):**
1.1. are screened and routinely evaluated for supportive care needs and unmet needs, followed by conversations with appropriate specialists or healthcare professionals towards effectively addressing these needs
1.2. receive survivorship care planning responsive to their clinical and personal needs that is regularly reviewed
1.3. receive survivorship care with consideration of person-reported experience and outcome measures as negotiated
1.4. are offered self-management strategies, self-management support, and education with consideration of their self-management capacity and health literacy
1.5. have their goals of care, life goals, and personal agency respected and supported through shared decision-making
1.6. have their financial needs evaluated, discussed, and addressed (where appropriate) throughout their care
***2. Coordinated and Integrated Care***
To provide people affected by advanced or metastatic cancer with continuity of care, coordination of care, and integration of health services (e.g., medical specialists, nursing, primary care, and allied health) across survivorship and palliative care phases, that facilitates efficient, innovative, and responsive ways of engaging the health workforce to optimally manage people affected by advanced or metastatic cancer
** People affected by advanced or metastatic cancer (i.e., cancer survivors, caregivers, and family members):**
2.1. are provided with patient navigation support to facilitate access to appropriate care and care coordination
2.2. receive early referrals to multidisciplinary and interprofessional supportive care services
2.3. are provided with a team-care approach between medical specialists, nursing, primary care, and allied health professionals
2.4. receive timely referral to specialist palliative care (depending on needs evaluated using palliative need assessment tools) for assessment, management or co-management from diagnosis
2.5. are offered models of care that best suit their needs and preferences (e.g., specialist-led, nurse-led, shared-care, primary care-led, supported self-management)
2.6. are offered a care plan to facilitate transition of care when there is a change in place of care or cancer center providing care
2.7. are offered models of peer support through support groups (online or face-to-face) and other community-led organisations
***3. Evidenced-Based and Comprehensive Care***
To provide up-to-date evidence-based clinical best practice and comprehensive supportive care programs for all people affected by advanced or metastatic cancer, that are informed and supported by ongoing professional development of health care professionals, and education programs delivered to cancer survivors, caregivers, administrators, and health care professionals
** People affected by advanced or metastatic cancer (i.e., cancer survivors, caregivers, and family members):**
3.1. receive care practices, innovations, and improvements that are translated from, and informed by research according to their local context in a culturally sensitive way
3.2. receive information on evidenced-based supportive care strategies to address their survivorship care needs
3.3. actively encouraged and supported in decision-making to promote health, manage disease, and reduce distress
3.4. receive multidisciplinary and interprofessional care that seeks to prevent or manage morbidities associated with cancer treatment
3.5. are treated by healthcare professionals (cancer specialists and non-cancer specialists) who integrate new evidence regarding supportive
3.6. care and issues into their practice through ongoing professional development and education
3.7. are treated as active contributors to the content of professional development and education materials for healthcare professionals
***4. Evaluated and Communicated Care***
To deliver routine and systematic evaluation and monitoring of supportive care needs, underpinned by established multi-lateral communication between all health care professionals, and people affected by advanced or metastatic cancer, that is timely, clear, effective, respectful, and appropriate (i.e., information and language suitable for the intended end-user), and facilitates conduct, delivery, and dissemination of clinical and supportive care evaluations to optimize quality survivorship care to people affected by advanced or metastatic cancer
** People affected by advanced or metastatic cancer (i.e., cancer survivors, caregivers, and family members):**
4.1. are systematically assessed and routinely re-assessed for supportive care interventions and referral (as required)
4.2. are supported with clear and timely communication processes, adopted by and between their healthcare providers
4.3. receive objective and subjective evaluations and monitoring of supportive care needs, outcomes, and experiences, that incorporate healthcare provider, cancer survivor, and caregiver perspectives
4.4. have secure medical records (electronic or paper-based) accessible on-demand by their specialists, primary care, and allied health, where appropriate
4.5. are embedded in healthcare settings that engage in service evaluations and quality improvement activities
***5. Accessible and Equitable Care***
To ensure models of cancer survivorship care are accessible (i.e., affordable, acceptable, available, and appropriate) and equitable for all people affected by advanced or metastatic cancer, so that quality of care does not vary because of personal factors (i.e., age, gender, geography, ethnicity, sexuality, language, physical or cognitive disability), cultural factors, or religious factors
** People affected by advanced or metastatic cancer (i.e., cancer survivors, caregivers, and family members):**
5.1. are offered, and provided, with consistent and high-quality survivorship care regardless of their personal factors
5.2. have their cultural needs acknowledged and respected within their supportive care, inclusive of language needs
5.3. have their spiritual needs acknowledged and respected within their supportive care, inclusive of religious beliefs
5.4. are offered care modalities and models that optimize accessibility and safety (i.e., telehealth, virtual, hybrid, face-to-face)
5.5. receive supportive care options that are innovative, inclusive, and targeted towards eliminating care disparities
5.6. are provided information about, and facilitated to connect with consumer groups, support networks, and organisations that advocate for accessible and equitable care
5.7. are supported by specified personnel within cancer centers and other care organisations (e.g., financial navigators or social workers) to access financial and legal assistance and guidance in financial literacy
***6. Sustainable and Resourced Care***
To ensure models of cancer survivorship care are sustainably designed and implemented to underpin high quality value-based care delivered in a cost-effective yet clinically meaningful manner for people affected by advanced or metastatic cancer. This includes the support for hospital and healthcare systems providing quality cancer survivorship care to be well-resourced (i.e., human resources, equipment, facilities, and leadership)
** People affected by advanced or metastatic cancer (i.e., cancer survivors, caregivers, and family members):**
6.1. receive value-based supportive care incorporating a stepped-care approach, matching intensity and acuity of needs and the level of care available and required
6.2. receive care in settings that are properly resourced to provide ongoing quality cancer survivorship care
6.3. receive supportive care from services that undergo routine evaluation and re-evaluation at all organizational levels
6.4. are embedded in healthcare settings with leadership that value, support, facilitate and invest in supportive care
6.5. receive appropriate quality supportive care using a resource-stratified approach
6.6. have access to care interventions and models that are clinically- and cost-effective within the local health context supported by adequate financial investment
7. ***Research and Data-Driven Care***
To provide quality and efficiency in cancer survivorship care for people affected by advanced or metastatic cancer through well-designed, and properly funded multidisciplinary research, together with established systems for local, national, and large-scale international data capture and information sharing through mutual informed consent. This seeks to optimize global capacity to share knowledge, data, and expertise that addresses unique and complex issues facing people affected by advanced or metastatic cancer
** People affected by advanced or metastatic cancer (i.e., cancer survivors, caregivers, and family members):**
7.1. are included in the co-design of clinical trials and research studies in cancer care
7.2. are included as participants of research trials focused on addressing cancer care
7.3. are informed of, and supported to access, all eligible and available clinical trials
7.4. are supported back to clinical and community care after completion or withdrawal from clinical trials
7.5. are evaluated using standardized cross-cultural tools (where available) to promote harmonized data capture and facilitate global data sharing and collaborations
7.6. have their experience, treatment, and outcome data routinely captured, and consistently reported and recorded
7.7. benefit from appropriate and equitable levels of financial and other investments into cancer care and survivorship research
7.8. can provide informed consent for, and facilitate having, their de-identified and harmonized supportive care data placed in data repositories for future research exploration and future health service improvement evaluations
**Additional Resources**
More information, including a supplement with additional evidence tables, slide sets, and clinical tools and resources, is available at www.mascc.org and www.asco.org/standards. Patient information is available at www.cancer.net

### Standards of survivorship care for people affected by advanced or metastatic cancer

#### Standard 1: person-centered care

Providing person-centered, survivorship care empowers people affected by advanced or metastatic cancer to have agency and be active partners in their care. This approach should facilitate active collaboration with trusted healthcare systems and practitioners who assess and respond to the unique needs of cancer survivors and caregivers respectively. Embedded within a person-centered approach, survivorship care and unmet needs must be regularly assessed and addressed as these fluctuate over time [4, 5, 11, 14, 24, 25, 28, 35, 36]. Areas of increased need for people affected by advanced or metastatic cancer are particularly identified in the financial, health system and information, psychological, and physical and daily living domains of supportive care [4, 5, 14, 24, 25, 36]. Survivorship care planning and discussions should use a written plan and ideally consider both clinical and personal needs and be regularly reviewed. This approach enhances adherence to care recommendations, communication with providers, and overall satisfaction with care [11–14, 20–22, 24, 25, 35, 37, 38]. While existing literature has not shown the necessary evidence that survivorship care plans improve clinical outcomes [39–41], cancer survivors and caregivers continue to value, and report benefit from such documents, supporting their use [42–44]. To facilitate person-centered care, a written survivorship care plan is considered fundamental for this subpopulation of cancer survivors that may have evolving treatment recommendations, treatment toxicities, and other healthcare needs that must be communicated in an ongoing fashion to multiple providers. Future research addressing the unique person-centered outcomes of survivorship care plans specifically for those with advanced or metastatic cancer is needed. In addition, self-management implemented with support and education that is tailored to capacity (including cognitive function; for example, among those with primary brain cancer or those with metastatic cancer to the brain) and health literacy is an additional powerful lever for achieving personalized high-quality cancer care, particularly for people with disease that requires complex and ever-changing regimens [11, 14, 17, 20, 25, 26, 37, 38, 45–47]. In the context of person-centered care for those with advanced or metastatic cancer, it is imperative to respect and support the goals and personal agency of survivors and their caregivers through a collaborative and shared decision-making process. Taking this approach ensures alignment with personal preference, despite having advanced or metastatic cancer, to have an active role in their cancer care and survivorship care provision [6, 11, 28, 35, 48, 49]. However, the fluidity of preferences must be taken into consideration for people who may be facing progression of disease, changes of treatment regimen and declines in overall health status and self-management capabilities. Hence, survivorship care should incorporate person-reported outcome and experience measures (PROMs and PREMs) that are routinely used to facilitate active cancer survivor and caregiver engagement and voices in shared decision-making and self-management strategies [11, 14, 24, 25, 28, 50, 51].

#### Standard 2: coordinated and integrated care

Continuity of care, care coordination, and the seamless integration of health services throughout survivorship phase are fundamental to best manage people affected by advanced or metastatic cancer. As care for these people may be more complex and involve additional healthcare providers in different disciplines and healthcare settings, providing patient navigation support is instrumental to facilitate access to appropriate and enduring care and care coordination through individualized assistance to overcome healthcare system barriers [21, 28, 29, 52–55]. Patient navigation not only improves cancer survivor and caregiver outcomes and satisfaction, but also yields economic and resource benefits, including reductions in emergency department visits, hospitalizations, and staff utilization [55]. An integrated team-care approach involving specialists, nursing, primary care, social workers and allied health with early referral to multidisciplinary and interprofessional supportive care services significantly improves the timely coordination and delivery of quality care [11, 12, 14, 20, 21, 24–26, 36, 49, 56, 57, 58]. This also includes timely referral to specialist palliative/end-of-life care based on needs, which is associated with improved quality of life, psychosocial outcomes, physical symptoms, and period of survival [19, 24–26, 48, 57, 58]. In addition, people affected by advanced or metastatic cancer often are, or feel, excluded from existing survivorship programs and resources; and may also not feel ‘celebrating survivorship’ resonates with their experiences; thus, should be offered models of peer support, such as support groups and other community-led organizations specifically targeting this patient population. These opportunities for informational support, shared experience, learning from others with similar experiences and disease trajectories, and cultivating coping strategies having positive impacts on psychological outcomes and personal empowerment [59, 60]. Lastly, different models of care have equivalent effectiveness for managing physical and psychosocial outcomes, and thus the model of care provided should be chosen based on the individual needs and preferences of people affected by advanced or metastatic cancer within various resource settings [12, 13, 17, 28, 36, 51, 61, 62]. In order to fulfill the needs of this population of cancer survivors and caregivers, building bridges and promoting close collaboration between survivorship, supportive care, and palliative care clinicians and programs is needed.

#### Standard 3: evidence-based and comprehensive care

People affected by advanced or metastatic cancer should not only receive the most up-to-date, evidence-based, best practice treatment, but also be provided with access to comprehensive survivorship care programs [11, 13, 14, 20, 25, 63] that continue to evolve their approach as guided by evidence. This dual approach ensures that care encompasses the broad spectrum of support required to address the multifaceted challenges experienced by people with advanced or metastatic cancer and their caregivers. Further, evidence-based and comprehensive care with multidisciplinary involvement plays a pivotal role in actively encouraging and supporting informed decision making, ultimately improving health and disease management as well as distress reduction [6, 11, 12, 14, 20, 25, 28, 48, 57]. It is also fundamental that evidence-based practice encompass culturally sensitive care and information on supportive care strategies that is not only derived from and informed by research, but also tailored to be relevant to the local context of those receiving care [6, 11, 25, 28, 35, 63]. People with different cultural backgrounds may manage uncertainty of their disease, referral to specialist palliative care, and ultimately end-of-life discussions differently [64, 65]. To facilitate continual best-practice, evidence-based care provision, it is also integral that survivorship care programs for people affected by advanced or metastatic cancer are informed by the ongoing professional development of healthcare professionals, with education programs to be delivered to all stakeholders [11, 13, 24–26, 29, 31, 63, 66]. Such education should include content and updates on the changing landscape of treatment and survival outcomes and cover the range of biopsychosocial issues people affected by advanced or metastatic cancer are prone to experience. Healthcare systems must address the significant barrier of resource allocation, ensuring that adequate resources are available to support staff in their ongoing educational pursuits [11, 13, 17, 24–26, 29, 31, 63, 66] particularly in addressing the needs and specifically inviting participation of and taking into account the perspectives of those with advanced or metastatic cancer. Their lived experiences enhance the relevance and effectiveness of educational material for the cancer survivors and their caregivers [4, 13, 17, 28, 29, 66, 67].

#### Standard 4: evaluated and communicated care

Ensuring the routine and systematic evaluation and monitoring of supportive care needs, along with appropriate referral to relevant survivorship care services and healthcare professionals, requires a foundation of established, multidirectional communication among all healthcare professionals and people affected by advanced or metastatic cancer [11, 12, 14, 17, 20, 21, 24–26]. This holistic evaluation approach aids identifying the full spectrum of challenges experienced by this population, and aids tailoring and delivering quality cancer survivorship care. Moreover, effective communication in this context should be timely, clear, effective, respectful, and appropriate. However, it is essential to acknowledge and address barriers that impede optimal communication in service provision, particularly for people with metastatic and advanced cancer and their caregivers, such as insufficient training in communication skills, time constraints, and inadequate systems to support eliciting and documenting communication [11, 14, 17, 20, 25, 51]. For example, while oncologists may be comfortable communicating with those with early-stage cancers and have had training to communicate with those nearing the end-of-life, it is important that there is additional training for clinicians to address survivorship care needs for people living with advanced or metastatic cancer. In addition to patient-clinician communication, to enable timely communication and collaboration between healthcare providers, secure medical records should also be accessible on demand [17, 21, 29, 68, 69]. Continual service evaluations and quality improvement activities, including the integration of emerging technologies such as artificial intelligence, should also be embedded in healthcare settings to optimize communication and care delivery [11, 13, 21, 63, 70]. Ongoing communication, as outlined earlier, with the entire health care is particularly crucial when treatment is complex, evolving and involves multiple clinical specialists and disciplines.

#### Standard 5: accessible and equitable care

Ensuring models of cancer survivorship care are not only comprehensive, but also accessible (i.e., affordable, acceptable, available, and appropriate) and equitable for all people affected by advanced or metastatic cancer is paramount to ensuring quality care does not vary due to personal, cultural, or religious factors [4, 13, 26, 28, 29, 35, 51, 53, 71, 72]. People with advanced or metastatic cancer are more likely to experience financial toxicity that may deter them from accessing care [73–75]. Health workforce diversity and cultural awareness training, as well as the development and provision of culturally and linguistically appropriate resources can empower healthcare professionals to better understand and cater to the unique needs of diverse cancer survivor and caregiver populations. Further, the development of health equity metrics for continual service evaluation and improvement can assist in ensuring supportive care options are innovative, inclusive, and targeted towards eliminating disparities [23, 28, 29, 51, 53, 71, 72]. It is essential to recognize that some people, particularly those with advanced or metastatic cancer, have increased spiritual support needs and when these needs are unmet, spiritual pain and distress can be exacerbated [4, 13, 26, 29, 35, 71, 72]. Furthermore, the design and choice of care modalities and care models should prioritize the optimization of accessibility and safety, utilizing telehealth, virtual clinics, and hybrid modes, each of which have been proven effective in optimizing cancer survivor outcomes while also potentially reducing costs, particularly for cancer survivors and caregivers in rural and remote communities [14, 29, 35, 51, 53, 70, 76–79]. Leveraging these approaches expands the reach of care and ensures that geographical barriers do not limit access to quality services, ultimately promoting equitable care. Finally, people affected by advanced or metastatic cancer who may face challenges and discrimination in fully or partially returning to work or previous work capacity, should also be supported to access consumer groups and support networks that actively advocate for accessible and equitable care as well as specific personnel within organizations to access employment, financial and legal assistance (e.g., financial navigators, social workers, or legal practitioners) [5, 11, 29, 53, 80–82].

#### Standard 6: sustainable and resourced care

Provision of ongoing high-quality cancer survivorship care for people affected by advanced or metastatic cancer necessitates a sustainable and adequately resourced approach. Supportive care should utilize a stepped-care and resource-stratified approach that first offers the least resource-intensive care that aligns with needs and available resources of the context of service provision and those receiving care. This need-based and value-based approach minimizes resource burden while also associated with notable reductions in distress and disease burden [25, 35, 49, 57, 69, 72, 83–86]. Survivorship care interventions and models for advanced or metastatic cancer should be cost-effective yet clinically meaningful and supported by adequate financial investment regardless of the types of health systems [11, 57, 61, 63, 70, 76, 87, 88]. Healthcare settings providing survivorship care need to be properly resourced to enable the provision of high-quality ongoing survivorship care for people affected by advanced or metastatic cancer. Intentional planning for support services across the cancer care continuum for those with metastatic and advanced cancer is essential and should be facilitated. In doing so, all relevant care settings (e.g. cancer centers, community) need to be provided with appropriate levels of human resources, equipment, facilities, as well as leadership who value, support, facilitate, and appropriately invest in survivorship care [11, 63, 86–88]. Continual evaluation of these services is important to ensure sustainability and to show positive return on investments to advocate for further investment in survivorship care for people affected by advanced or metastatic cancer [11, 54, 60, 67, 84–86].

#### Standard 7: research and data-driven care

Well-designed and properly funded multidisciplinary research is integral to improving the quality and efficiency of cancer survivorship care for people affected by advanced or metastatic cancer [6, 8, 17, 23, 24, 28, 29]. To enable population-wide surveillance of the incidence and prevalence of people with advanced or metastatic cancer (de novo metastatic or recurrent disease progression), cancer registries must be established or expanded [1, 89–92]. Active involvement of people affected by advanced cancer in the co-design of research is critical to better meet the needs of end-users and enhance the rigor, relevance, reach, and impact of survivorship research [5–8, 23, 24, 28, 29, 93, 94]. To enhance accessibility of high-quality survivorship care for this population, research trials should broaden eligibility criteria to explicitly include people affected by advanced or metastatic cancer in clinical trial design and proposed survivorship care research questions, rather than focusing solely on earlier stage disease post-curative-intent treatment; address barriers to enrolment and participation at all system levels; and consider the potential use of patient navigators or other facilitators needed to promote knowledge of, entry into, and transition out of cancer clinical trials as appropriate [7, 28, 29, 67, 94]. To facilitate harmonized data capture, global collaborative sharing of data, and use of data repositories for future research; it is important to constantly and accurately record experience, treatment, and outcome data in clinical trials using standardized cross-cultural assessment tools, where available [4, 6, 17, 20, 23, 24, 28, 29, 67, 70, 72, 95–100]. Another critical factor to consider in advanced or metastatic cancer survivorship research is how participants are supported in their transition back to clinical and community care following clinical trial participation. Participants transitioning out of cancer trials have reported heightened symptoms and emotions as well as salient awareness of their limited remaining life span and care options [7, 80, 94, 101, 102]. To enhance survivorship care for people affected by advanced or metastatic cancer, high-quality coordinated research is required. Ensuring appropriate and equitable investments into advanced or metastatic cancer survivorship research is crucial, highlighting the ongoing need for continual funding opportunities in this specific population of cancer survivors and caregivers [6, 23, 24, 28, 29].

## Discussion

In response to calls for improved advanced or metastatic cancer survivorship care, this joint MASCC-ASCO effort developed standards and practice recommendations for the consistent provision of quality survivorship care of people with advanced or metastatic cancer and their caregivers. Using a comprehensive and systematic process, seven standards comprising 45 practice recommendations were developed. These standards provide guidance to key stakeholders including healthcare providers and professionals; healthcare leadership, administrators, and managers; governments, health ministries, and policymakers; as well as cancer survivors and caregivers. These standards enable these stakeholders, in various contexts, to plan, prepare, provide, resource, fund, and advocate for the delivery of high-quality survivorship care for people affected by advanced or metastatic cancer. Further, these standards and practice recommendations are consistent with, and complementary to, other existing ASCO standards and guidelines that are listed under the Related ASCO Guidelines and Standards section.

There has been tremendous progress made in the recognition of quality cancer survivorship care, leading to the improvements in clinical care, growing research, development of specialized programs, and ongoing advocacy for policy changes. However, much of this effort has centered around cancer survivors who have completed cancer treatment. The unique and often complex cancer survivorship needs of those living with advanced or metastatic cancer have not been adequately addressed. Recognizing disparate health systems, availability of resources, and workforce capacities worldwide, these MASCC-ASCO standards and practice recommendations for survivorship care were developed to address existing gaps and to help promote clinical care, research and policy specifically targeting this growing population of people affected by advanced or metastatic cancer. Indeed, the standards and recommendations outlined are designed to support local planning and implementation efforts within various resource constraints by allowing prioritization within the local context of the stakeholder, and through facilitating identifiable areas for investment, development, future directives, and decisive actions by key stakeholders. Clinical resources should be developed to support the integration of these standards and practice recommendations by stakeholders, including clinician toolkits, continuing medical education, training, and other resources [13, 15, 17]. Global dissemination, implementation, and evaluation of the MASCC-ASCO standards and practice recommendations for quality survivorship care of people affected by advanced or metastatic cancer will benefit from establishing resource-stratification frameworks, clinician and cancer survivor materials for use and engagement (promoting patient-clinician communication in accordance with ASCO’s consensus guideline [103]), as well as language translations and cultural adaptations. This ensures the MASCC-ASCO standards can be utilized across regions around the world and thus guide advanced or metastatic survivorship care and research globally.

In conclusion, the MASCC-ASCO standards have been developed to promote provision of high quality, evidence-based survivorship care for people with advanced or metastatic cancer and their caregivers, emphasizing the need for care that is person-centered, coordinated and integrated, evidence-based and comprehensive, evaluated and communicated, accessible and equitable, sustainable and resourced, as well as research and data-driven. These standards and practice recommendations provide a critical resource to healthcare stakeholders in order to facilitate tailored and effective advanced or metastatic cancer survivorship care across disciplines and settings. Given the ever-changing landscape of treatments and disease trajectories over time, in settings where advanced cancer is less clearly defined (e.g., hematological or central nervous system malignancies), it is important that users of these MASCC-ASCO standards understand the contextual challenges experienced by these less defined advanced cancer populations, and appropriately apply survivorship care standards for people affected by advanced or metastatic cancer, rather than excluding them.

**Table:** 

RELATED ASCO GUIDELINES, STANDARDS, AND STATEMENTS
**ASCO Guidelines** • Practical Assessment and Management of Vulnerabilities in Older Patients [104] (http://ascopubs.org/doi/10.1200/JCO.23.00933) • Palliative Care in the Global Setting Resource-Stratified [105] (http://ascopubs.org/doi/10.1200/JGO.18.00026) • Integration of Palliative Care into Standard Oncology Practice [106] (http://ascopubs.org/doi/10.1200/JCO.2016.70.1474) • Patient-Clinician Communication [103] (http://ascopubs.org/doi/10.1200/JCO.2017.75.2311) • Management of Anxiety and Depression in Adult Survivors of Cancer [107] (https://ascopubs.org/doi/full/10.1200/JCO.23.00293) • Screening, Assessment, and Management of Fatigue in Adult Survivors of Cancer [108] (https://ascopubs.org/doi/full/10.1200/jco.2013.53.4495) • Management of Chronic Pain in Survivors of Adult Cancers [109] (https://ascopubs.org/doi/full/10.1200/JCO.2016.68.5206)
**ASCO Standards** • Oncology Medical Home Standards [110] (http://ascopubs.org/doi/10.1200/OP.21.00167) • Telehealth Standards in Oncology [78] (http://ascopubs.org/doi/10.1200/OP.21.00438)
**ASCO Statements** • Achieving High-Quality Cancer Survivorship Care [13] (https://ascopubs.org/doi/10.1200/JCO.2012.46.6854) • Toward Individualized Care for Patients with Advanced Care [17] (https://ascopubs.org/doi/10.1200/JCO.2010.33.1744)

### Supplementary Information

Below is the link to the electronic supplementary material.

Supplementary file1 (PDF 391 KB)

Supplementary file2 (PDF 33.7 KB)

Supplementary file3 (PDF 23.4 KB)

## Data Availability

All data is provided as Supplementary Files - therefore all of our data is available to all people as required.

## Declarations

NHH and JY are MASCC ‘Terry Langbaum’ Survivorship Fellows. NHH is MASCC Chair of Fatigue and MASCC Chair of Exercise Oncology. RJC, MIF, and MJ are MASCC Chair and Vice-Chairs Survivorship. GBC and SM are MASCC Chair and Vice-Chair of Palliative Care. BK is MASCC Chair of Psychosocial. RJC is also a MASCC Executive Board Member. MBL is MASCC President. FDA is a MASCC Ex-Officio Member. RZ is ASCO President (2024–2025). NHH and RJC receive salary support from the National Health and Medical Research Council as Investigator Fellows (APP2017080 and APP1194051). This article was also prepared as part of MAM official duties as an employee of the US Federal Government. The findings and conclusions in this report are those of the authorship and do not necessarily represent the official position of the National Cancer Institute.
