# Supplementary material for: Survivorship care for people affected by advanced or metastatic cancer: MASCC-ASCO standards and practice recommendations
Source: Support Care Cancer. 2024 Apr 29;32(5):313. doi: 10.1007/s00520-024-08465-8 (PMC11056340; doi:10.1007/s00520-024-08465-8)
Supplement: Supplementary file 1 — Supplementary file1 (PDF 391 KB) [file 520_2024_8465_MOESM1_ESM.docx]

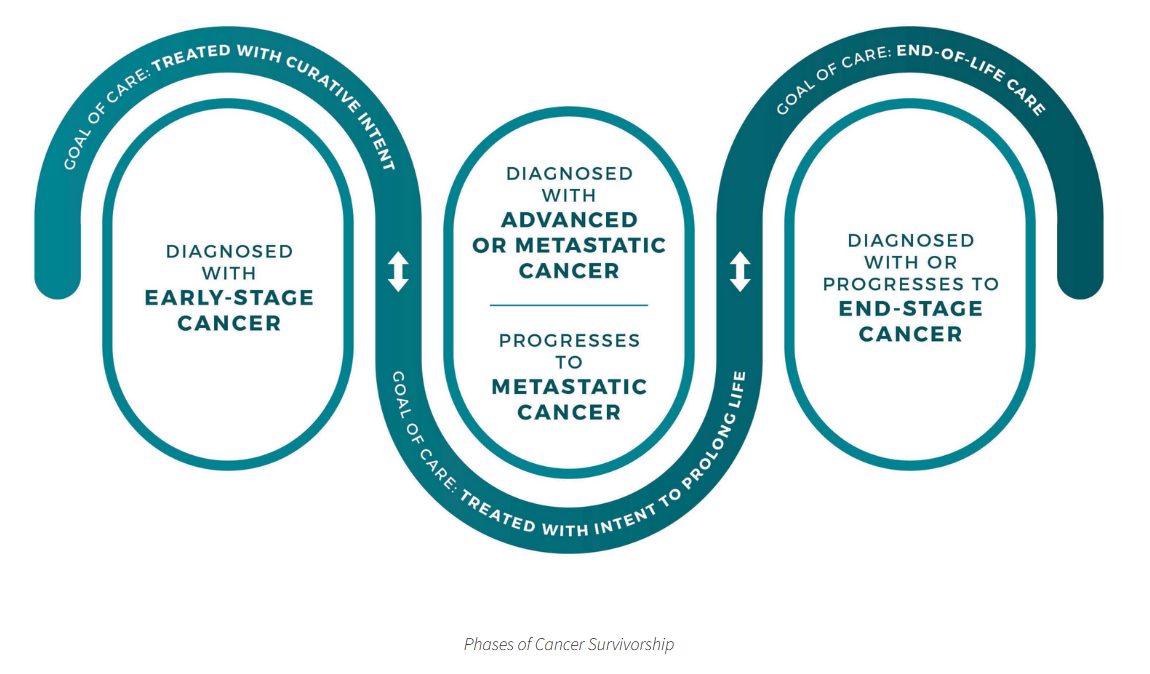


Appendix 1: Phases of Cancer Survivorship (a) early-stage cancer treated with curative intent, (b) advanced or metastatic cancer treated with intent to prolong life, and (c) end-stage cancer provided with end-of-life care. These MASCC-ASCO standards are focused on the advanced or metastatic cancer phase of cancer survivorship [6].
