# Supplementary material for: Survivorship care for people affected by advanced or metastatic cancer: MASCC-ASCO standards and practice recommendations
Source: Support Care Cancer. 2024 Apr 29;32(5):313. doi: 10.1007/s00520-024-08465-8 (PMC11056340; doi:10.1007/s00520-024-08465-8)
Supplement: Supplementary file 2 — Supplementary file2 (PDF 33.7 KB) [file 520_2024_8465_MOESM2_ESM.docx]

***Appendix 2.* Demographic characteristics of panel experts across the modified Delphi consensus process.**

|  | **Round 1 (n=77)** | | **Round 2 (n=51)** | |
| --- | --- | --- | --- | --- |
|  | **N** | **%** | **N** | **%** |
| **Age** | | | | |
| - Years | 45.7 (Mean) | ± 11.7 (SD) | 44.9 (Mean) | ± 10.5 (SD) |
| **Gender** | | | | |
| - Female | 45 | 58.4% | 30 | 58.8% |
| - Male | 32 | 41.6% | 21 | 41.1% |
| **Race or Ethnicity** | | | | |
| - Asian | 11 | 14.3% | 8 | 15.7% |
| - Biracial or Multiracial | 2 | 2.6% | 1 | 2.0% |
| - Black, African, or African American | 4 | 5.2% | 2 | 4.0% |
| - Caucasian | 54 | 70.1% | 38 | 76.3% |
| - Hispanic or Latino | 6 | 7.8% | 2 | 4.0% |
| **WHO Region^a^** **where person or professional has lived or worked with advanced and metastatic cancer** | | | | |
| - African Region (AFR) | 3 | 3.9% | 3 | 5.9% |
| - Region of the Americas (AMR) | 31 | 40.3% | 20 | 39.2% |
| - South-East Asian Region SEAR) | 5 | 6.5% | 2 | 3.9% |
| - European Region (EUR) | 49 | 63.6% | 31 | 60.8% |
| - Eastern Mediterranean Region (EMR) | 6 | 7.8% | 3 | 5.9% |
| - Western Pacific Region (WPR) | 28 | 36.4% | 22 | 43.1% |
| **World Bank categorisation of income for countries represented by participants** | | | | |
| - High-income countries | 22 | 66.8% | 17 | 71.0% |
| - Upper-middle income countries | 7 | 21.1% | 5 | 20.8% |
| - Lower-middle income countries | 3 | 9.1% | 1 | 4.1% |
| - Low-income countries | 1 | 3% | 1 | 4.1% |
| **Role in Cancer Care** | | | | |
| - **Patient Advocate** | **8** | **10.4%** | **6** | **11.8%** |
| - - *Cancer Survivor* | *6* | *7.8%* | *4* | *7.8%* |
| - - *Caregiver* | *2* | *2.6%* | *2* | *3.9%* |
| - **Academic or Researcher** | **24** | **31.2%** | **24** | **47.0%** |
| - **Allied Health Professional** | **18** | **23.4%** | **16** | **31.4%** |
| - - *Dietitian or Nutritionist* | *3* | *3.9%* | *3* | *5.9%* |
| - - *Exercise Physiologist* | *8* | *10.4%* | *7* | *13.7%* |
| - - *Physical Therapist* | *3* | *3.9%* | *3* | *5.9%* |
| - - *Psychologist* | *3* | *3.9%* | *2* | *3.9%* |
| - - *Social Worker* | *1* | *1.3%* | *1* | *2.0%* |
| - **Cancer Specialists** | **45** | **58.4%** | **27** | **47.0%** |
| - - *Surgeon* | *1* | *1.3%* | *1* | *2.0%* |
| - - *Hematologist* | *1* | *1.3%* | *1* | *2.0%* |
| - - *Medical Oncologist* | *18* | *23.4%* | *10* | *19.6%* |
| - - *Radiation Oncologist* | *1* | *1.3%* | *1* | *2.0%* |
| - - *Palliative Care Physician* | *11* | *14.3%* | *7* | *13.7%* |
| - - *Primary Care Physician* | *3* | *3.9%* | *3* | *5.9%* |
| - - *Cancer Nurse* | *10* | *13.0%* | *4* | *7.8%* |
| - **Other (unspecified)** | **11** | **14.3%** | **5** | **9.8%** |
| **Experience (Years) with Advanced/Metastatic** | | | | |
| - 0 to 4 years | 8 | 10.8% | 6 | 11.8% |
| - 5 to 9 years | 18 | 24.3% | 10 | 19.6% |
| - 10 to 14 years | 19 | 25.7% | 13 | 25.4% |
| - 15 to 20 years | 11 | 14.9% | 11 | 21.6% |
| - 20 years or more | 18 | 24.3% | 11 | 21.6% |
| **Highest Education** | | | | |
| - Bachelor’s Degree | 3 | 3.9% | 2 | 3.9% |
| - Doctor of Medicine (MBBS or MD) | 28 | 36.4% | 19 | 37.2% |
| - Doctor of Philosophy (PhD) | 36 | 46.8% | 25 | 49.0% |
| - High School | 1 | 1.3% | 0 | 0.0% |
| - Master’s Degree | 9 | 11.7% | 5 | 9.8% |

a = Countries of the world separated into regions in accordance with the World Health Organization: African Region (Ghana, Nigeria, and Rwanda), Region of the Americas (Brazil, Canada, Mexico, and the United States of America), South-East Asian Region (India and Thailand), European Region (Belgium, Denmark, France, Germany, Iceland, Ireland, Italy, Netherlands, Poland, Portugal, Spain, Sweden, Switzerland, Turkey, and the United Kingdom), Eastern Mediterranean Region (Bahrain, Kuwait, Saudi Arabia, and United Arab Emirates), and Western Pacific Region (Australia, China, Japan, New Zealand, and Taiwan).

**MASCC-ASCO Survivorship Care for People affected by Advanced or Metastatic Cancer Expert Panel**

| **Name (and Designation)** | **Affiliation or Institution** | **Area of Expertise** |
| --- | --- | --- |
| Nicolas H. Hart PhD, AES, CSCS, FESSA (Co-Chair) | Human Performance Research Centre, INSIGHT Research Institute, University of Technology Sydney (UTS), Sydney, NSW, Australia | Cancer Survivorship,  Allied Health |
| Raymond J. Chan RN, PhD, FAAN, FACN, GAICD (Co-Chair) | Caring Futures Institute, College of Nursing and Health Sciences, Flinders University, Adelaide, SA, Australia | Cancer Survivorship,  Cancer Nursing |
| Larissa Nekhlyudov MD, MPH, FASCO | Brigham and Women’s Hospital and Dana-Farber Cancer Institute, Boston, MA, United States of America | Cancer Survivorship,  Primary Care |
| Thomas J. Smith MD, FACP, FASCO, FAAHPM | Sidney Kimmel Comprehensive Cancer Center, The John Hopkins Hospital, Baltimore, MD, United States of America | Medical Oncology,  Patient Advocate |
| Margaret I. Fitch RN, PhD | School of Graduate Studies, Faculty of Nursing, University of Toronto, Toronto, ON, Canada | Cancer Nursing |
| Gregory B. Crawford AM, MBBS, MD, FRACGP, FAChPM | Faculty of Health and Medical Sciences, University of Adelaide, Adelaide, SA, Australia | Palliative Care, Primary Care |
| Meera R. Agar PhD, MPC, MBBS, FRACP, FAChPM | IMPACCT Research Centre, INSIGHT Research Institute University of Technology Sydney (UTS), Sydney, NSW, Australia | Palliative Care |
| Bogda Koczwara AM, MBBS MBioethics, FRACP, FAICD | Flinders Health and Medical Research Institute, College of Medicine and Public Health, Flinders University, Adelaide, SA, Australia | Cancer Survivorship,  Medical Oncology |
| Sandip Mukhopadhyay MD, MBBS | Indian Council of Medical Research, National Institute of Cholera and Enteric Diseases, Kolkata, India | Palliative Care |
| Jasmine Yee PhD, AEP | School of Psychology, Faculty of Science, The University of Sydney, Sydney, NSW, Australia. | Cancer Survivorship,  Allied Health |
| Frederick D. Ashbury PhD, MACE | Department of Oncology, University of Calgary, Calgary, AB, Canada | Supportive Care |
| Maryam B. Lustberg MD, MPH | Smilow Cancer Hospital and Yale Cancer Center, New Haven, CT, United States of America | Medical Oncology |
| Michelle Mollica RN, PhD, OCN | Division of Cancer Control and Population Sciences, National Cancer Institute, Bethesda, MD, United States of America | Cancer Survivorship |
| Andrea L. Smith PhD | The Daffodil Centre and The University of Sydney, Sydney, NSW, Australia | Cancer Survivorship, Patient Advocate |
| Michael Jefford MBBS, PhD, MPH | Australian Cancer Survivorship Centre and Peter MacCallum Cancer Centre, University of Melbourne, Melbourne, Australia | Cancer Survivorship,  Medical Oncology |
| Fumiko Chino MD | Memorial Sloan Kettering Cancer Center, New York, NY, United States of America | Radiation Oncology |
| Robin Zon MD, FACP, FASCO | Michiana Hematology Oncology; Memorial Hospital, Mishawaka, IN, United States of America | Medical Oncology |
