# Supplementary material for: Survivorship care for people affected by advanced or metastatic cancer: MASCC-ASCO standards and practice recommendations
Source: Support Care Cancer. 2024 Apr 29;32(5):313. doi: 10.1007/s00520-024-08465-8 (PMC11056340; doi:10.1007/s00520-024-08465-8)
Supplement: Supplementary file 3 — Supplementary file3 (PDF 23.4 KB) [file 520_2024_8465_MOESM3_ESM.docx]

*Appendix 3*. Consensus results for endorsed Standards and Recommendations of Survivorship Care for People affected by Advanced or Metastatic Cancer.

| **MASCC-ASCO Quality Survivorship Care Standards** | **Agreement  (%)^a^** | **Likert Score  (Mean (SD))^b^** |
| --- | --- | --- |
| **STANDARD 1: PERSON CENTERED CARE**  To recognize people affected by advanced or metastatic cancer as individuals with agency and partners in cancer care, who are served by, and participate in, trusted health systems that respond to their unique needs (e.g., physical, psychosocial, health systems, information, financial, fertility, sexual, spiritual, and relationships) in humane and holistic ways in collaboration with health practitioners and health care organizations in the public, private and not-for-profit health and related sectors. | 96.4% | 4.74 (0.60) |
| **RECOMMENDATIONS:**  People affected by advanced or metastatic cancer (i.e., cancer survivors, caregivers, and family members):   - 1. are screened and routinely evaluated for supportive care needs and unmet needs, followed by conversations with appropriate specialists or healthcare professionals towards effectively addressing these needs.   2. receive survivorship care planning responsive to their clinical and personal needs that is regularly reviewed.   3. receive survivorship care with consideration of person reported experience and outcome measures as negotiated.   4. are offered self-management strategies, self-management support, and education with consideration of their self-management capacity and health literacy.   5. have their goals of care, life goals, and personal agency respected and supported through shared decision-making.   6. have their financial needs evaluated, discussed, and addressed (where appropriate) through-out their care. | 91.3%  86.0%  87.3%  92.2%  93.5%  88.3% | 4.59 (0.85)  4.33 (0.90)  4.44 (0.78)  4.59 (0.70)  4.67 (0.71)  4.43 (0.84) |
| **STANDARD 2: COORDINATED AND INTEGRATED CARE**  To provide people affected by advanced or metastatic cancer with continuity of care, coordination of care, and integration of health  services (e.g., medical specialists, nursing, primary care, and allied health) across survivorship and palliative care phases, that facilitates  efficient, innovative, and responsive ways of engaging the health workforce to optimally manage people affected by advanced or metastatic cancer. | 95.1% | 4.73 (0.66) |
| **RECOMMENDATIONS:**  People affected by advanced or metastatic cancer (i.e., cancer survivors, caregivers, and family members):   - 1. are provided with patient navigation support to facilitate access to appropriate care and care coordination.   2. receive early referral to multidisciplinary and interprofessional supportive care services.   3. are provided with a team-care approach between medical specialists, nursing, primary care, and allied health professionals.   4. receive timely referral to specialist palliative care (depending on needs evaluated using palliative need assessment tools) for assessment, management or co-management from diagnosis.   5. are offered models of care that best suit their needs and preferences (e.g., specialist-led, nurse-led, shared-care, primary care-led, supported self-management).   6. are offered a care plan to facilitate transition of care when there is a change in place of care or cancer center providing care.   7. are offered models of peer support through support groups (online or face-to-face) and other community-led organisations | 95.4%  91.1%  93.3%  87.3%  87.3%  84.2%  86.7% | 4.53 (0.72)  4.51 (0.91)  4.47 (0.88)  4.44 (0.71)  4.51 (0.84)  4.27 (0.99)  4.29 (0.98) |
| **STANDARD 3: EVIDENCE-BASED AND COMPREHENSIVE CARE**  To provide up-to-date evidence-based clinical best practice and comprehensive supportive care programs for all people affected by advanced or metastatic cancer, that are informed and supported by ongoing professional development of health care professionals, and education programs delivered to cancer survivors, caregivers, administrators, and health care professionals. | 97.4% | 4.77 (0.63) |
| **RECOMMENDATIONS:** People affected by advanced or metastatic cancer (i.e., cancer survivors, caregivers, and family members):   - 1. receive care practices, innovations, and improvements that are translated from, and informed by research according to their local context in a culturally sensitive way.   2. receive information on evidenced-based supportive care strategies to address their survivorship care needs.   3. actively encouraged and supported in decision-making to promote health, manage disease, and reduce distress.   4. receive multidisciplinary and interprofessional care that seeks to prevent or manage morbidities associated with cancer treatment.   5. are treated by healthcare professionals (cancer specialists and non-cancer specialists) who integrate new evidence regarding supportive care and issues into their practice through ongoing professional development and education.   6. are treated as active contributors to the content of professional development and education materials for healthcare professionals. | 96.1%  97.5%  94.8%  96.2%  90.0%  88.4% | 4.67 (0.59)  4.73 (0.50)  4.49 (0.84)  4.73 (0.52)  4.66 (0.67)  4.35 (0.95) |
| **STANDARD 4: EVALUATED AND COMMUNICATED CARE**  To deliver routine and systematic evaluation and monitoring of supportive care needs, underpinned by established multi-lateral communication between all health care professionals, and people affected by advanced or metastatic cancer, that is timely, clear, effective, respectful, and appropriate (i.e., information and language suitable for the intended end-user), and facilitates conduct, delivery, and dissemination of clinical and supportive care evaluations to optimize quality survivorship care to people affected by advanced or metastatic cancer. | 94.8% | 4.63 (0.71) |
| **RECOMMENDATIONS:** People affected by advanced or metastatic cancer (i.e., cancer survivors, caregivers, and family members):   - 1. are systematically assessed and routinely re-assessed for supportive care interventions and referral (as required).   2. are supported with clear and timely communication processes, adopted by and between their healthcare providers.   3. receive objective and subjective evaluations and monitoring of supportive care needs, outcomes, and experiences, that incorporate healthcare provider, cancer survivor, and caregiver perspectives.   4. have secure medical records (electronic or paper-based) accessible on-demand by their specialists, primary care, and allied health, where appropriate.   5. are embedded in healthcare settings that engage in service evaluations and quality improvement activities. | 92.2%  91.0%  91.0%  88.9%  88.3% | 4.56 (0.67)  4.58 (0.73)  4.54 (0.73)  4.47 (0.81)  4.60 (0.74) |
| **STANDARD 5: ACCESSIBLE AND EQUITABLE CARE**  To ensure models of cancer survivorship care are accessible (i.e., affordable, acceptable, available, and appropriate) and equitable for all people affected by advanced or metastatic cancer, so that quality of care does not vary because of personal factors (i.e., age, gender, geography, ethnicity, sexuality, language, physical or cognitive disability), cultural factors, or religious factors. | 96.1% | 4.68 (0.73) |
| **RECOMMENDATIONS:**  People affected by advanced or metastatic cancer (i.e., cancer survivors, caregivers, and family members):   - 1. are offered, and provided, with consistent and high-quality survivorship care regardless of their personal factors.   2. have their cultural needs acknowledged and respected within their supportive care, inclusive of language needs.   3. have their spiritual needs acknowledged and respected within their supportive care, inclusive of religious beliefs.   4. are offered care modalities and models that optimize accessibility and safety (i.e., telehealth, virtual clinics, hybrid, face-to-face).   5. receive supportive care options that are innovative, inclusive, and targeted towards eliminating care disparities.   6. are provided information about, and facilitated to connect with consumer groups, support networks, and organisations that advocate for accessible and equitable care.   7. are supported by specified personnel within cancer centers and other care organisations (e.g., financial navigators or social workers) to access financial and legal assistance and guidance in financial literacy. | 93.5%  94.8%  90.9%  92.2%  93.5%  84.4%  90.6% | 4.67 (0.67)  4.67 (0.57)  4.60 (0.69)  4.62 (0.70)  4.68 (0.59)  4.35 (0.80)  4.53 (0.81) |
| **STANDARD 6: SUSTAINABLE AND RESOURCED CARE**  To ensure models of cancer survivorship care are sustainably designed and implemented to underpin high quality value-based care delivered in a cost-effective yet clinically meaningful manner for people affected by advanced or metastatic cancer. This includes the support for hospital and healthcare systems providing quality cancer survivorship care to be well-resourced (i.e., human resources, equipment, facilities, and leadership). | 96.1% | 4.66 (0.65) |
| **RECOMMENDATIONS:**  People affected by advanced or metastatic cancer (i.e., cancer survivors, caregivers, and family members):   - 1. receive value-based supportive cancer care incorporating a stepped-care approach, matching intensity and acuity of needs and the level of care available and required.   2. receive care in settings that are properly resourced to provide ongoing quality cancer survivorship care.   3. receive supportive care from services that undergo routine evaluation and re-evaluation at all organizational levels.   4. are embedded in healthcare settings with leadership that value, support, facilitate and invest in supportive care.   5. receive appropriate quality supportive care using a resource-stratified approach.   6. have access to care interventions and models that are clinically- and cost-effective within the local health context supported by adequate financial investment. | 92.2%  93.5%  94.8%  92.2%  93.5%  93.6% | 4.47 (0.67)  4.56 (0.72)  4.53 (0.71)  4.53 (0.80)  4.67 (0.67)  4.63 (0.72) |
| **STANDARD 7: RESEARCH AND DATA-DRIVEN CARE**  To provide quality and efficiency in cancer survivorship care for people affected by advanced or metastatic cancer through well- designed, and properly funded multidisciplinary research, together with established systems for local, national, and large-scale international data capture and information sharing through mutual informed consent. This seeks to optimize global capacity to share knowledge, data, and expertise that addresses unique and complex issues facing people affected by advanced or metastatic cancer. | 94.8% | 4.65 (0.76) |
| **RECOMMENDATIONS:**  People affected by advanced or metastatic cancer (i.e., cancer survivors, caregivers, and family members):   - 1. are included in the co-design of clinical trials and research studies in cancer care.   2. are included as participants of research trials focused on addressing cancer care.   3. are informed of, and supported to access, all eligible and available clinical trials.   4. are supported back to clinical and community care after completion or withdrawal from clinical trials.   5. are evaluated using standardized cross-cultural tools (where available) to promote harmonized data capture and facilitate global data sharing and collaborations.   6. have their experience, treatment, and outcome data routinely captured, and consistently reported and recorded.   7. benefit from appropriate and equitable levels of financial and other investments into cancer care and survivorship research.   8. can provide informed consent for, and facilitate having, their de-identified and harmonized supportive care data placed in data repositories for future research exploration and future health service improvement evaluations. | 89.8%  93.5%  92.2%  90.9%  92.2%  92.2%  84.4%  93.5% | 4.54 (0.75)  4.62 (0.68)  4.63 (0.62)  4.54 (0.81)  4.56 (0.67)  4.54 (0.71)  4.38 (0.97)  4.55 (0.61) |

**Note:** a = agreement level reported as the percentage sum of agree and strongly agree votes per statement; b = Likert Score reported as the mean and standard deviation of ranking produced within the 5-point Likert scale, where 4 is Agree and 5 is Strongly Agree.
